# GENDER, SEXUAL ORIENTATION, AND SPOUSAL CAREGIVING FOR THOSE WITH DEMENTIA: A FOCUS ON EMOTION WORK

**DOI:** 10.1093/geroni/igad104.1732

**Published:** 2023-12-21

**Authors:** Toni Calasanti, Sadie Giles, Jing Geng

**Affiliations:** Virginia Tech, Blacksburg, Virginia, United States; Virginia Tech, Blacksburg, Virginia, United States; Virginia Tech, Blacksburg, Virginia, United States

## Abstract

This study draws upon and expands three research streams. The first relates to spousal caregiving for those with dementia, and gender differences in stress. Based on gender divisions of labor, men are more likely to take a task-oriented approach to caregiving; women take a more holistic, empathetic approach. The second strand relates to emotion work, wherein caregivers manipulate their own emotions to produce a desired outcome in another (Hochschild, 2003). Research shows that spouses find performing emotion work “draining” (Umberson, et al., 2020), and that women do it more. Finally, we draw upon research that finds that same-sex couples are more egalitarian in their division of labor than are straight couples; how this impacts caregiving approaches or emotion work is unclear. Weaving these together, our study explores how inequalities of gender and sexuality intersect in shaping caregiving by older adults who care for their demented spouses. Our analysis of in-depth interviews conducted among 69 straight, gay, and lesbian caregivers finds that, first, heterosexual men rarely engage in emotion work, though they do manage their own feelings. Second, heterosexual wives overwhelmingly manipulate their feelings in attempts to gain compliance from their spouses, calm them, and/or make them happy. Finally, both gay men and lesbians perform emotion work, and they tend to combine the task-oriented and holistic approaches to care. These findings make clear that gender differences found in previous caregiving research are not “natural,” and suggest that straight men’s lower stress levels may result from their tendency to skip emotion work.

